# Metabolomics in epidemiology: from metabolite concentrations to integrative reaction networks

**DOI:** 10.1093/ije/dyw046

**Published:** 2016-04-26

**Authors:** Liam G Fearnley, Michael Inouye

**Affiliations:** ^1^Centre for Systems Genomics; ^2^School of BioSciences; ^3^Department of Pathology, University of Melbourne, Parkville, VIC, Australia

**Keywords:** systems biology, epidemiology, metabolomics, transcriptomics, genomics

## Abstract

Metabolomics is becoming feasible for population-scale studies of human disease. In this review, we survey epidemiological studies that leverage metabolomics and multi-omics to gain insight into disease mechanisms. We outline key practical, technological and analytical limitations while also highlighting recent successes in integrating these data. The use of multi-omics to infer reaction rates is discussed as a potential future direction for metabolomics research, as a means of identifying biomarkers as well as inferring causality. Furthermore, we highlight established analysis approaches as well as simulation-based methods currently used in single- and multi-cell levels in systems biology.

Key Messages

- Recent advances in high-throughput technologies now allow generation of population-scale metabolomics and other ‘omics’ data.
- Parallel advances in computational and statistical approaches enable the integration of these data.
- Consequently, analytical approaches that consider single concentration-based metabolites can now integrate additional omics data and existing databases to build reaction network models, which may give more insight into human disease.

## Introduction

Biological research has traditionally been investigated through reductionist approaches, in part due to limitations in both experimental measurement and analytical sophistication.^1^ The recent development of high-throughput systems-wide technologies has led to a dramatic increase in the number of quantifiable properties at the organismal, cellular and molecular levels.^2^ Whereas reductionist approaches can be applied to the data generated from these technological innovations, doing so either ignores potentially important patterns in the data or incurs inefficiency in the application of such technologies.^3^^,^^4^ Research questions answered using high-throughput technologies have required a parallel conceptual shift in data analysis. This transition from the common manual analysis of single measurements and simple mixtures to the probing of systems-level behaviour using more sophisticated computational and statistical methods characterizes modern biology.

There is thus a complex interaction at play between modern systems-level technologies, epidemiology and analytical methods. Many biological systems contain large numbers of relevant variables (typically hundreds to millions) and harbour no small amount of sampling and non-biological variation. Therefore, to gather enough observations per variable and attain adequate study power, there is a need for the population-scale study of these systems, and epidemiological approaches have a substantial role to play. However, rigorous epidemiological application places extraordinary demands on systems-level technologies, primarily in terms of maximal throughput, accuracy and cost-effectiveness.

Sixty years after A.T. James and A.J.P. Martin pioneered the use of gas chromatography and mass spectrometry to separate and detect individual volatile fatty acids,^5^ and 40 years after Hoult *et al.*’s measurements of tissue metabolites using ^31^P nuclear magnetic resonance spectroscopy,^6^ the many current forms of mass spectrometry (MS) and nuclear magnetic resonance (NMR) spectroscopy are routinely and widely used to measure far greater numbers of identifiable metabolites across much longer time periods and at far less cost.^7^^,^^8^ MS techniques based on flow injection are now capable of measuring thousands of samples per day while measuring a broad swathe of metabolites.^9–11^ In general, MS-based analyses are subject to a number of limitations. Certain metabolites cannot be captured because of to the physical processes involved, and identification of spectra utilizes libraries of spectral standards, which can be particularly problematic to do in a rapid, automated and accurate way if spectra contain noise. Most MS methods are also destructive in nature, given the requirement for separation, ionization, fragmentation and acceleration of the sample’s components through a magnetic field. NMR spectrometry is a complementary approach which compensates for some of MS’s limitations. Modern NMR metabolomics platforms can perform high throughput, accurate measurement of standard biomarkers at less cost than MS;^12^ however coverage of metabolites is not as complete, since convoluted NMR signals and spectra make quantification of some individual metabolites difficult.

For the application of metabolomics to epidemiology, accurate quantitation, speed of processing and cost are key barriers. These technical challenges are being rapidly addressed for both NMR- and MS-based approaches; however, epidemiological studies of the metabolome have largely been restricted to easily and ethically accessible tissues. These primarily include blood serum/plasma, urine, faecal material and saliva. However, a large portion of metabolism occurs in difficult-to-assay tissues, such as the liver, gut and kidneys, which are central to pathology, energy generation and drug metabolism. Therefore, a key technical frontier for metabolomics will be either direct metabolite quantification or inference in these tissues.

Other systems-level technologies have experienced dramatic advances in recent years. In genomics, first-generation sequencing methods enabled the sequencing of the human genome^13^ and, two decades later, next-generation methods are routinely capable of the sequencing a human genome in hours at a cost of roughly $1,000. Measurement of other features including the epigenome,^14^ transcriptome,^15^ proteome^16^^,^^17^ and other ‘omics’^18^^,^^19^ have undergone similar trajectories in this time frame, allowing researchers to begin defining disease using characteristics at the molecular rather than physiological level (e.g. in breast cancer^20^^,^^21^ and familial hypercholesterolaemia^22^).

**Figure. 1.**
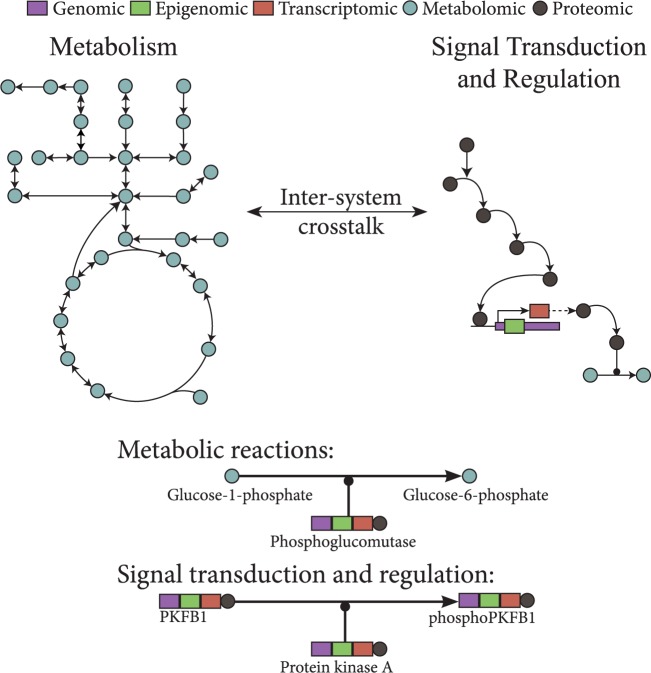
A schematic of integrating metabolomics data with other multi-omics data as part of known reaction networks**.** Metabolomics together with other multi-omics data can be integrated into the analysis of metabolism at different points in various systems. Genomic, epigenomic, transcriptomic and proteomic variation all have various direct and indirect effects on the function and cross-talk of various metabolic and signalling networks.

Consequently, computational and statistical approaches to omics data which treat cells, tissues and organs as whole, integrated systems rather than isolated individual processes are required, both in the study of individual organisms and in the study of populations. Here, we review and discuss two areas of intense research in the application of quantitative metabolomics to epidemiology, broadly divided into advances in statistical approaches which integrate multi-omics data (Figure 1) and techniques that extract and leverage reaction rate information.

## Integrating metabolomics and genomics

Quantitative MS and NMR metabolomics approaches currently provide data for a range of statistical analyses, including standard association-based testing, multivariate analyses and metabolite set enrichment techniques to emerging techniques of pathway and whole-systems level analysis.

Metabolomic association studies, which aim to establish an association between a metabolite(s) and a particular condition or quantitative trait, have proved a valuable approach for biomarker identification and the metabolic underpinnings of disease,^23–25^ but can be limited by statistical power issues related to sample sizes and coverage of metabolites.^26^ Gaussian Graphical Models (GGMs), which evaluate conditional dependencies in multivariate Gaussian distributions, are one method for analysing quantitative metabolic data. Since many metabolites are well characterized in terms of their role in reactions, GGMs can be used to predict previously unknown or unannotated reactions from single-time-point metabolic data.^27^^,^^28^ This allows reconstruction of metabolic pathways with or without prior knowledge—a potentially useful technique for identifying candidate determinants of abnormal metabolism and its downstream consequences. Another technique, metabolite set enrichment,^29^ is based on the widely-used gene set enrichment technique (GSEA).^30^ Both metabolite and gene set enrichment use prior knowledge about genes involved in cellular processes. These sets of genes are either manually annotated, as in the Gene Ontology,^31^ or derived from pathway databases such as the Kyoto Encyclopedia of Genomes and Genes (KEGG).^32^ Sets of metabolites are used to generate scores which can be compared between conditions to determine differentially enriched processes.^33^ Overall, set enrichment methods are useful in assessing and interpreting change due to cumulative effects where phenotype is altered by low-magnitude changes across metabolites.

Rapid advances in human genomics have led to the widespread use of genome-wide association studies (GWAS) to identify genetic variants which affect downstream phenotype.^34^ A metabolomic GWAS, where each sample has paired genome-wide genotype and metabolomic data, aims to detect genetic loci associated with variation in metabolic phenotype.^35–40^ In metabolomic GWAS, metabolite concentrations are tested for association with individual genetic variants using standard statistical approaches, such as linear regression, together with stringent significance levels which correct for substantial multiple testing burdens. Metabolomic GWAS have been immensely successful in population-based cohorts to gain insight into the genomics of serum lipid and small organic metabolites.^41^^,^^42^ An exemplar is the KORA F4 study^43^ which used fasting serum metabolomics for various genome-wide association studies of metabolic traits.^44–46^ Many metabolic trait loci were located in or near genes encoding enzymes mediating rate-limiting steps in a number of metabolic reactions.^44^ Importantly, estimated effect sizes for associated genetic variants were relatively high, likely due to the testing of specific metabolites which have well defined roles in metabolic pathways, rather than agglomerated ‘total' metabolites.^45^ Furthermore, many metabolite loci have been reported as associated with drug toxicity^45^ or complex diseases, such as that of *SLC22A4* with Crohn’s disease.^47–49^

Extending these genetic approaches to capture causal relationships between metabolites and (molecular) traits or diseases is possible through techniques exploiting mendelian randomization (MR). MR uses genetic variants as instrumental variables for testing for casual relationships. The distributions of these variables are relatively free of environmental confounding factors, as they are assigned randomly from parental genotypes during the formation of gametes.^50^ Classic MR techniques assume that these instrumental variables are free of the influence of factors that confound the association of the metabolite of interest and the putative outcome, and that the variable chosen must be associated with the exposure. These assumptions are stronger for assessing the causal effects of epigenetic variation,^51^ but two-step MR techniques can address this shortcoming by treating epigenetic variation itself as an intermediate phenotype. This approach and its extensions can be readily applied to metabolic variation^52^ and, potentially, where metabolic outcomes in turn modify epigenetic state.^53^^,^^54^ For epidemiological studies of human disease, MR has been used to investigate the roles of total high-density lipoprotein (HDL) and low-density lipoprotein (LDL) cholesterol in heart disease,^55–57^ the causal effects of exposures on metabolites^58^ and in testing whether changes to metabolites affect disease risk.^59^ MR can also be exploited to determine causal relationships at the reaction or pathway level^60^ as well as to study more complex combinations of multiple phenotypes.^61^

Metabolomics has had a significant impact on next-generation sequencing studies of human microbiota. The existence of host-microbiota interactions is well established,^62^^,^^63^ and the composition of the microbiome plays a role in many diseases including obesity,^64^ asthma^65^ and diabetes.^66^ A potentially important point-of-effect lies at the interface of microbial and host metabolomes, which is known to be an important conduit for molecular exchange,^67^ and advances in quantitative metabolomics have allowed researchers to trace metabolic activity from substrate input (e.g. in the host diet) through the host-microbe metabolic interface and on to associated changes in disease risk.^68^

## The metabolome-transcriptome interface

**Figure. 2.**
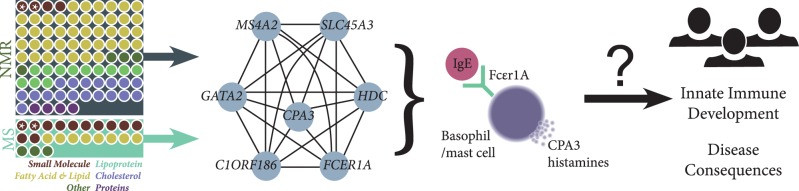
The lipid-leukocyte (LL) module and its known metabolite associations. A number of classes of metabolites (left, via NMR^77^ and MS^78^) are associated with the LL co-expression module. Starred metabolites (leucine and isoleucine) are directly quantified on both MS and NMR platforms. This module is expressed in basophils and mast cells, which play significant roles in both disease and the development of the innate immune system. The specific role of the LL module in these processes remains unknown.

Quantitative metabolomics data and the inferred function of metabolic pathways largely depend on the level and function of specific enzymes, which are in turn controlled by transcription of specific genes. Gene transcription is a complex yet tightly regulated process. Reconstruction of transcriptional networks has long been an area of intense research,^69–74^ but the relationship between the transcriptome and metabolome remains a largely unexplored area. Epidemiological cohorts and the omics profiling of their corresponding biospecimens have played a key role in elucidating this interface.^75^ Studies of gene co-expression networks and their associations with serum metabolomic profiles have revealed the existence of a gene module, the lipid leukocyte (LL) module, which appears to be associated with and responsive to diverse metabolite concentrations (Figure 2).^76^^,^^77^ The genes contained within the module encode enzymes and proteins with functions indicative of basophil- and mast cell-mediated immune response.^77^ Whereas it has been shown to potentially play a wide role in metabolism,^77^ the LL module was originally identified through associations with *APOB*, total HDL and triglyceride levels.^76^ In addition, individual gene transcript analysis identified carnitine palmitoyl transferase A1 (*CPTA1*) and carnitine/acylcarnitine translocase (*SLC25A20*) associations with circulating free fatty acids.^76^ Carnitine transferase also featured in a recent landmark investigation of the metabolome-transcriptome interface in the KORA F4 study.^78^ This study generated a pathway-level interaction network of gene ontologies and metabolic pathways, together with transcription factor binding enrichment analysis, to identify diverse regulatory interactions, network motifs and signatures associated with HDL cholesterol and triglyceride levels.^78^ The same study also replicated the co-expression and diverse metabolic relationships of the core LL module. Systems-level association studies of this kind represent a minimally biased approach toward the discovery of key interaction points between metabolism and gene transcription; however, an important area of future investigation is the reduction of these systems-level associations into mechanistic studies of single genes and single metabolites in relevant *in vivo* and *in vitro* contexts. At the same time, epidemiological cohorts can be further leveraged to characterize the extensive cross-talk and condition-specific interactions of these systems, thus further guiding mechanistic studies.

## Reaction rates as biomarkers

The epidemiological study of disease has increasingly come to focus on the use of metabolite concentrations as biomarkers^79^ which are themselves commonly used as proxies for metabolic reaction rates.^44^^,^^80^ However, assessment of rates of individual reactions may provide stronger markers of trait or disease.

Direct measurement of metabolic reaction rates *in situ* is currently impractical in large population studies but has been achieved on smaller scales, most notably through the use of non-invasive NMR spectroscopy.^81^ Although such studies are also expensive, technically challenging and require significant infrastructure, they suggest that reaction rates (or metabolic flux) can serve as stronger biomarkers than metabolite concentrations. Metabolic flux imaging techniques using hyperpolarized metabolites have shown promise in the diagnosis and localization of tumours in prostate cancer patients,^82^ and a number of studies have investigated reaction fluxes in the cardiovascular systems of model organisms.^83^^,^^84^ An epidemiological study of particular note is a prospective study in a set of 58 heart failure patients where the investigators measured the rate of ATP synthesis through cardiac creatine kinase flux *in situ* using ^31^P magnetic resonance spectroscopy.^85^ ATP and creatine phosphate concentrations as well as common clinical scores were used as predictors of heart failure over an 8.2 year follow-up period. Abnormal creatine kinase flux significantly outperformed patient age, gender and metabolite concentrations in predicting heart failure events and death, including hospitalization for heart failure, cardiac mortality, cardiac transplantation and ventricular-assist device placement, as well as all-cause mortality.^85^^,^^86^ These results are in a relatively small patient cohort with a limited number of events, but they add weight to the argument for the development of reaction rate-based biomarkers in the study of disease.

**Figure. 3.**
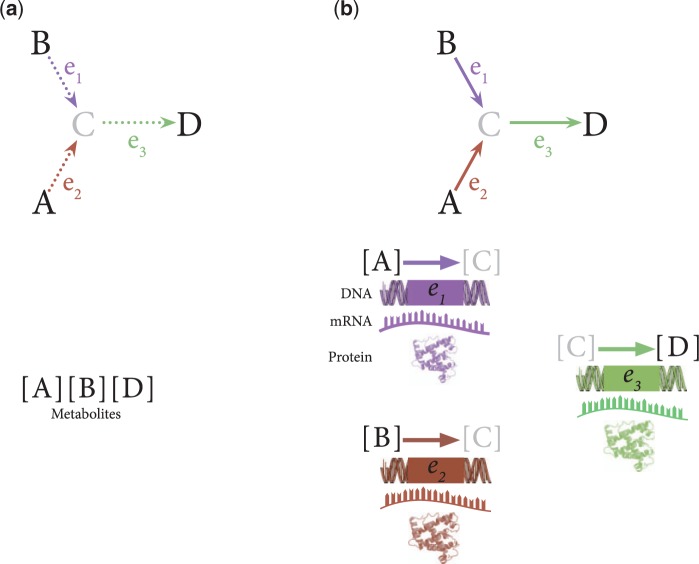
Steady-state metabolic measurements vs integrative multi-system measurement and modelling. a) Single-time, steady-state metabolic measurement directly measures the concentration of metabolites within the metabolism, but does not provide direct insight into the known interactions between these molecules. b) Integrative, multi-system measurement and modelling provide this missing insight into the interactions within the system; not only are metabolites measured, but also information about the rates of reactions that convert metabolites into other metabolites are inferred, modelled or predicted, providing more insight into the behaviour of the system as a whole.

Conceptually, metabolism behaves like a system in which molecules ‘flow’ through reactions. As the flow of metabolites is blocked and re-routed, metabolites accumulate at various points or are depleted, with resulting changes in their concentration. Metabolite concentrations capture the effects of combined changes to reaction rates, but do not provide direct insight into the processes themselves, for example the pathogenic variation affecting enzymes, genes and other molecular products derived from the organism's genome (Figure 3a). As noted above, direct measurement of enzyme function and other key mediators of reaction rates is immensely challenging *in situ* due to expense and technical difficulty. *In vitro* assays face additional challenges including sometimes prohibitive requirements for the quantity and type of tissue required, and technical bias introduced by adaptation of cells to the culture environment.

**Figure. 4.**
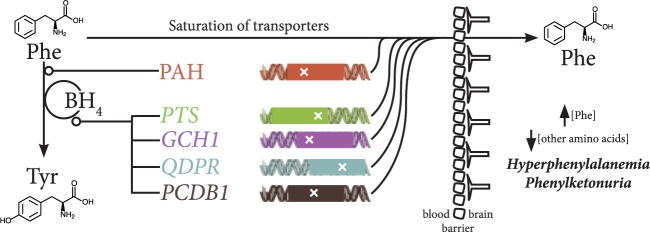
An overview of the molecular and genetic basis of phenylketonuria (PKU). PKU is an inborn error of metabolism where symptoms occur due to accumulation of phenylalanine (Phe) in the blood (hyperphenylalanaemia), which overwhelms transporters that carry amino acids over the blood-brain barrier.

Systematic assessment of reaction rates at scales required for epidemiology might occur through the integration of metabolomic data with genomic, transcriptomic and/or proteomic information to infer enzymatic function, with subsequent comparison across conditions to determine where bottlenecks occur (Figure 3b). An initial step toward large-scale characterization of enzymatic function might leverage public reference panels for loss-of-function (LoF) variants.^87^ An individual with a gene encoding an enzyme with an LoF variant, such as a premature stop codon, will be at reduced or nil capacity to perform a specific biochemical reaction or set of reactions. This information can be used to build predictive models of the reaction system that have ramifications for metabolism (Figure 3b). For example, phenylketonuria (PKU) is a metabolic disease characterized by intellectual disability, microcephaly and seizures, and whose cause is genetic (Figure 3). Individuals with PKU inherit genetic variants that prevent the conversion of phenylalanine to tyrosine either through loss-of-function of phenylalanine hydroxylase (PAH) or an enzymatic cofactor, biopterin (BH4). The latter occurs through mutations to any of four genes (*PTS*, *GCH1*, *QDPR*, *PCDB1*) encoding subunits of enzymes catalyzing biopterin recycling. These mutations cause the build-up of phenylalanine which overwhelms transporters that carry amino acids across the blood–brain barrier, thus increasing phenylalanine concentration and reducing concentration of other amino acids in the brain during critical stages of development (Figure 4).^88^ Whereas metabolomic screening may be useful in identification of PKU, the integration of genomic data in particular offers a clear advantage towards characterization of reaction rates and timely identification of causal effects thereof. For complex diseases, such as cardiovascular disease, systems-level perturbations in metabolomic and genomic variation, as well as their integration with further omics information, will likely be required to build useful models of reaction rate variation and their pathogenesis.

## Inference of reaction rate at scale

The overwhelming majority of metabolite concentration data available are single-time-point tissue samples from large cohorts or high-resolution time courses for small groups of individuals. Extracting information on reaction networks can be challenging; however, two techniques which show promise are statistical analyses incorporating metabolite ratios and simulation of genome-scale metabolic flux models. Pathway-based analyses are often proposed as an alternative technique, but they contain inherent bias because: (i) pathways vary widely between databases and the expertise used to construct them; and (ii) databases and corresponding analyses typically treat pathways as separate entities, thus largely ignoring the inherent cross-talk between pathways^33^ which is a common feature of metabolism and implicated in many diseases.^89–91^

One way to try to extract information about the processes using metabolite concentrations is to use metabolite ratios (e.g. the ratio of the concentration of phenylalanine to that of tyrosine in PKU patients). This technique is relatively commonplace in the study of drug metabolism. Historically, ratios have been used to discriminate between multiple reactions acting on the same substrate or as a measure of drug clearance.^92^^,^^93^ They can act as an accurate proxy for the direct measurement of reaction rates within a region of the broader metabolic network, given certain assumptions about the metabolic state.^46^^,^^80^ A key hurdle for the analysis of metabolite ratios lies in their selection from the immense number of variables for assessment. The RECON2 model of human metabolism describes 2626 unique metabolites and thus 3 446 625 possible pair-wise combinations in metabolite ratios.^94^ Unfortunately, the majority of these ratios are biologically uninformative. The P-gain statistic, which calculates the increase in information contained within a metabolite ratio relative to the individual concentrations, has been widely used as a method for reducing this to a manageable number.^95^ Despite these limitations, initial work has been performed using metabolite ratios as traits in genome-wide scans for genetic loci involved in metabolism, with some success.^45^^,^^96^

Integration of metabolomics with genomic, transcriptomic and proteomic data from large cohorts and case-control studies offers systems-level characterization of the key factors in biochemical reactions. These 'genome-scale' models of metabolism are widely used in the bioengineering and systems biology communities as a tool for computational hypothesis generation and testing.^97^ They are derived from community-generated sets of known reactions (such as KEGG^32^ or Reactome^98^), which are then parameterized and fitted to experimental measurements for simulation and analysis. This parameterization and fitting process routinely incorporates genomic and transcriptomic information. In humans, the RECON2^94^ model is one such comprehensive set of metabolic data and parameters. The resulting models can then be analysed using various techniques, including constraint-based reconstruction and analysis (COBRA),^97^^,^^99^ optimization-based approaches and a host of other simulation methods.^100–102^ Comparing individualized instances of these metabolic models for each patient in a large cohort could yield valuable information about the downstream effects of genomic variation and subsequent processing of metabolites. However, many challenges remain. Such *in silico* experiments are extremely computationally expensive, particularly when they span multiple cellular processes. Even if a computational model is available, a key conceptual problem lies in determining and testing the relevant environmental conditions and stimuli required in order to generate the disease’s symptoms—for example, PKU symptoms do not appear unless the individual consumes excess phenylalanine (and a phenylalanine-free diet is indeed the current treatment strategy for individuals with these genetic variants). Developing strategies to overcome this problem represents one of the main conceptual hurdles to such analyses, and population-based studies will be important in adequate sampling and molecular characterization of conditions and sub-groups relevant to pathogenesis.

**Table 1.** Summary of measurement technologies and analysis techniques discussed in this review with selected example references

Measurement technologies	Description
Mass spectrometry	Rapid detection of low-concentration metabolites^103^
GC-MS	Separation of volatile metabolites^104^
LC-MS	Separation of non-volatile metabolites, broad scope^104^
Direct infusion	Fast broad coverage of metabolites^105^
High-throughput NMR	Complementary measurement technology; precise concentration measurement^106^
Metabolic flux imaging	*In situ* measurement of reaction rates in patients; non-invasive and non-destructive^81^

**Analysis techniques**	**Description**

Metabolic association studies	Direct analogue of GWAS studies; testing of metabolites for association with phenotype^24^
Gaussian graphical modelling	Inference and reconstruction of metabolic pathways where reactions are unknown^27^
Pathway analysis	Test for enrichment of sets of functionally related entities associated with phenotype^33^
Gene set enrichment analysis	Gene sets sourced from databases and ontologies (e.g. Gene Ontology)^30^
Metabolic set enrichment analysis	Metabolite sets sourced from databases (e.g. KEGG database)^29^
Metabolomic GWAS	Finding single nucleotide polymorphism s (SNPs) correlated with metabolic markers; GWAS with metabolite as trait^42^
Classic mendelian randomization	Determination of causal relationships between an exposure and outcome of interest using SNP as instrument^55^
Two-step MR	As for classic MR, but enables the testing of intermediate phenotypes that may confound the instrument^107^
Metabolite association with co-expression networks	Association of metabolite measurements with systems of genes that have similar expression behaviour^76^
Metabolite ratios	Association of ratios of metabolites, used as proxies for reaction rates, with a phenotype^45^
Genome-scale model simulation	Simulation of known reactions incorporating genetic variation^97^

## Conclusions

A key challenge for integrative metabolomics analysis at the population level lies in the development and standardization of analytical techniques which are routinely applied to large-scale datasets. Complete metabolic models will require a conceptual shift from metabolite concentrations towards experiments and graph-theoretical analyses based on the reactions themselves. At present, such approaches are largely applied in laboratory-based experiments of cell lines, but observational studies in patient- and population-level cohorts are becoming more common, thus enabling the identification of sub-groups of individuals enriched for variation in relevant sub-regions of the reaction network. Extraction and exploitation of reaction rates from quantitative metabolomics together with integration with other biomolecular systems data remains a key challenge but a promising future direction for molecular epidemiology.

## Funding

LGF and MI were supported by the National Health and Medical Research Council (NHMRC) of Australia (grant no. 1062227). MI was also supported by a Career Development Fellowship co-funded by the NHMRC and the National Heart Foundation of Australia (no. 1061435).

**Conflicts of interest:** None.
